# Cocaine-related cervical spinal cord infarction: a case report and review of the literature

**DOI:** 10.1186/s13256-021-03223-4

**Published:** 2022-02-02

**Authors:** F. Pichiorri, M. Masciullo, C. Foti, M. Molinari, G. Scivoletto

**Affiliations:** 1 Spinal Cord Unit, IRCCS S. Lucia Foundation, Via Ardeatina 306, 00179 Rome, Italy; 2 SPInal REhabilitation Lab (SPIRE), IRCCS Fondazione Santa Lucia, Rome, Italy; 3 Physical and Rehabilitation Medicine, University of Rome “Tor Vergata”, Rome, Italy

**Keywords:** Cocaine, Spinal cord ischemia, Anterior spinal artery, “Pencil-like” lesion, Case report

## Abstract

**Study design:**

Case report.

**Objectives:**

To report a clinical case of spinal cord infarction due to cocaine use.

**Setting:**

Spinal Center, IRCCS Fondazione S. Lucia, Rome (Italy).

**Case presentation:**

Two days after recreational use of cocaine, a 27-year-old Caucasic man was admitted to the emergency department for acute cervical pain, weakness in all four limbs, and urinary retention. A cervical spinal magnetic resonance imaging scan, performed after 2 days, showed a “pencil-like” lesion extending from C4 to T1 metamer, compatible with acute ischemia in the anterior spinal artery territory. Other causes of vascular disorders, as well as inflammatory and infectious disorders were ruled out. At admission in our department, the patient had an incomplete tetraplegia at level C6, an indwelling catheter, and was unable to stand and walk. After 3 months of rehabilitation, he had an AIS score D tetraplegia at level C7, was able to stand and walk using parallel bars, and indwelling catheter was replaced by intermittent catheterization.

**Discussion and conclusions:**

The etiology of medullary infarction may remain unexplained in nearly 30–40% of cases. Even if rare, cocaine-induced ischemic myelopathy should be considered and ruled out in the differential diagnosis of any acute nontraumatic myelopathy, especially in young patients.

## Introduction

The use of recreational drugs is a rising phenomenon, with an estimated prevalence of 5% in the global population aged 15–64 years [1].

Cocaine is one of the most abused illicit drug, especially among young adults between the second and fourth decade of life, and is responsible for many neurologic complications. These are typically related to the vascular effects at intracranial level [2], nonetheless few cases may result in acute spinal cord ischemia syndrome (ASCIS) [3–8].

## Case presentation

**Fig. 1:**
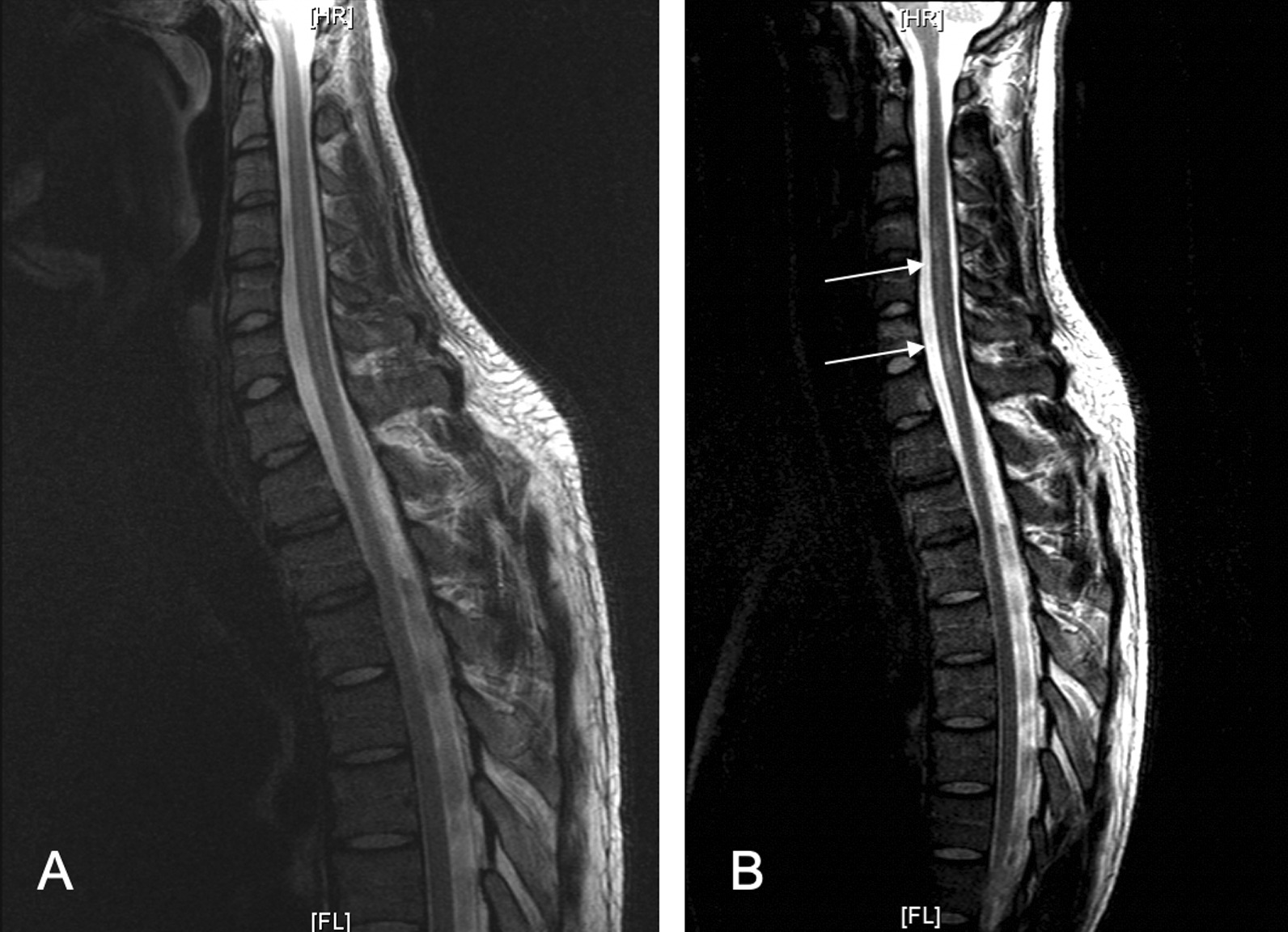
**A** Initial MRI of the spinal cord, which does not show any lesion. **B** MRI of the spinal cord 2 days after the onset of symptoms. The sagittal image shows a “pencil-like” hyperintensity extending from C4 to T1 metamer (white arrows)

Two days after recreational use of cocaine (via nasal sniffing), a 27-year-old Caucasic man was admitted to the emergency department for acute cervical pain, followed after a few hours by progressive weakness in all four limbs, and urinary retention. The neurologic examination showed a flaccid tetraparesis (grade 3 out of 5), with sensitivity impairment below C7–T1 level. The Glasgow Coma scale at admission was 15. The spinal cord magnetic resonance imaging (MRI) was normal (Fig. 1A), and the electrophysiological examination excluded a neuromuscular disorder. Motor evoked potentials (MEP) showed the absence of evoked motor responses through maximal cortical and cervical stimulation for thenar recordings; a normal motor response for tibialis anterior recordings was instead evoked only through the lumbar stimulation. In the emergency ward, vital signs were as follows: blood pressure (BP) 125/75 mmHg, heart rate (HR) 74 beats per minute, oxygen saturation (SaO_2_) 98%, and temperature (*T*) 36 °C. Routine blood tests and electrolytes were as follows: white blood cells 8400/mm^3^, red blood cells 4.61 million/mm^3^, hemoglobin 12.8 g/dL, hematocrit 39.10%; platelets 472,000/mm^3^, prothrombin activity 85.45%, international normalized ratio 1.13, blood urea 44 mg/dL, creatinine 0.71 mg/dL, blood glucose 99 mg/dL, sodium 135.0 mmol/L, potassium 4.5 mmol/L, and C-reactive protein 4.3 mg/L.

Thrombophilia and vasculitic screening, chest radiography, echocardiography, abdomen sonography, and extracranial ultrasound were normal. Infections, malignancies, malabsorption, and autoimmune disorders were excluded on both serum and cerebrospinal fluid.

The patient underwent corticosteroids and antiplatelet agents therapy (cardioaspirine and low-molecular-weight heparin)

A new cervical spinal MRI (after 2 days) showed a “pencil-like” hyperintensity extending from C4 to T1 metamer in sagittal T2-weighted images (Fig. 1B), compatible with acute ischemia in the anterior spinal artery (ASA) territory, associated with a swelling of the spinal cord.

At admission in our spinal center, the patient had an incomplete tetraplegia at level C6 and a strength of 3/5 on the Medical Research Council scale in all segments, with a more severe impairment of the right side. He was unable to stand and walk, had an indwelling catheter, and was severely dependent in daily life activities. At discharge, after 3 months of rehabilitation, he showed marked neurological and functional improvement. He shows an incomplete tetraplegia at level C7, and a strength of 3–4 in all segments. He is completely independent in daily life activities through the use of a wheelchair, and is able to stand and walk using the parallel bars. The indwelling catheter has been removed, and the patients is practicing clean intermittent catheterization.

## Discussion and conclusions

Cocaine abuse is well known to be associated with cerebrovascular events, but a few cases of cocaine-induced acute spinal cord ischemia (ASCIS) have also been described [3–8]. All involved young adults (age range 19–37 years), and in all of them the lesion was mainly located in the ASA territory of the cervical spinal cord (between C2 and T2) [4–6, 8], except one case [7] involving T1–T4 levels and one case with involvement of the posterior spinal artery at cervical level (C2) [3].

The exact mechanisms of cocaine-related vascular events may be direct or indirect and include [1]:

- Vasoconstriction and disruption of blood flow autoregulation in the nervous system
- Increased risk of vascular thrombosis, related to an altered platelet aggregation
- Cardio-embolism related to cocaine-induced myocardial infarction or cardiomyopathy

The most common MRI finding is a “pencil-like” hyperintensity in sagittal T2-weighted images [9], mainly located in the ASA territory at cervical level. Despite the good collateral supply in the spinal cord, which may explain the lower frequency of medullary infarction, a lack of blood supply from the ASA is more likely to provoke an anterior cord syndrome at cervical level because of insufficient anastomoses for higher perfused regions in the upper cervical part [3–5].

Despite severe neurological deficits, MRI of the spinal cord may not be able to detect any lesion in the first hours or days [5], while neurophysiological findings may be altered since the beginning. In particular, MEPs may help to localize the level of central motor pathway dysfunction [4].

Because of the few data available, the outcome in these patients remains unclear. As for other traumatic and nontraumatic spinal cord ischemia (SCIs), the rehabilitative outcome of patients with cocaine-related ASCIS may not be determined by the etiology of the lesion: in fact, both neurological and functional outcomes seem to depend on AIS grade and lesion level, and (possibly) age [10]. Radiologically, the outcome is worse when the lesion involves more than two spinal cord metamers [3–5].

ASCIS is a frequent cause of spinal cord lesion and accounts for about 25% of the nontraumatic lesions; 27% of patients are below 50 years of age (unpublished data). In about 30% of patients, clear vascular risk factors may not be detected and the etiology of ASCIS is not identified [10]. As there is nothing distinctive in cocaine-related ASCIS compared with the other most common forms of ASCIS, this etiology should be ruled out in case of any acute nontraumatic myelopathy, especially in younger patients.

## Data Availability

Data available upon reasonable request.
